# Supplementary material for: A fault-intrusion-tolerant system and deadline-aware algorithm for scheduling scientific workflow in the cloud
Source: PeerJ Comput Sci. 2021 Nov 2;7:e747. doi: 10.7717/peerj-cs.747 (PMC8576554; doi:10.7717/peerj-cs.747)
Supplement: Supplemental Information 6 [file peerj-cs-07-747-s006.zip › Myproject1.1/docs/org/cloudbus/cloudsim/network/package-summary.html]

org.cloudbus.cloudsim.network


JavaScript is disabled on your browser.


- Overview
- Package
- Class
- Use
- Tree
- Deprecated
- Index
- Help

- Prev Package
- Next Package

- Frames
- No Frames

- All Classes

# Package org.cloudbus.cloudsim.network

- Interface Summary

  | Interface | Description |
  | --- | --- |
  |  |  |
  | --- | --- |
  | GraphReaderIF | This interface abstracts an reader for different graph-file-formats |
- Class Summary

  | Class | Description |
  | --- | --- |
  |  |  |
  | --- | --- |
  | DelayMatrix\_Float | This class represents an delay-topology storing every distance between connected nodes |
  | FloydWarshall\_Float | FloydWarshall algorithm to calculate all pairs delay and predecessor matrix. |
  | GraphReaderBrite | This class is just an file-reader for the special brite-format! the brite-file is structured as followed: Node-section: NodeID, xpos, ypos, indegree, outdegree, ASid, type(router/AS) Edge-section: EdgeID, fromNode, toNode, euclideanLength, linkDelay, linkBandwith, AS\_from, AS\_to, type |
  | TopologicalGraph | This class represents an graph containing nodes and edges, used for input with an network-layer Graphical-Output Restricions! EdgeColors: GraphicalProperties.getColorEdge NodeColors: GraphicalProperties.getColorNode |
  | TopologicalLink | This class represents an link (edge) from an graph |
  | TopologicalNode | Just represents an topological network node retrieves its information from an topological-generated file (eg. |

- Overview
- Package
- Class
- Use
- Tree
- Deprecated
- Index
- Help

- Prev Package
- Next Package

- Frames
- No Frames

- All Classes
